# Prenatal Exposure to Methamphetamine: Up-Regulation of Brain Receptor Genes

**DOI:** 10.3389/fnins.2019.00771

**Published:** 2019-08-01

**Authors:** Hana Zoubková, Anežka Tomášková, Kateryna Nohejlová, Marie Černá, Romana Šlamberová

**Affiliations:** ^1^Department of Medical Genetics, Third Faculty of Medicine, Charles University, Prague, Czechia; ^2^Department of Physiology, Third Faculty of Medicine, Charles University, Prague, Czechia

**Keywords:** prenatal, methamphetamine, striatum, hippocampus, prefrontal cortex, receptor, microarray, real-time PCR

## Abstract

Methamphetamine (METH) is a widespread illicit drug. If it is taken by pregnant women, it passes through the placenta and just as it affects the mother, it can impair the development of the offspring. The aim of our study was to identify candidates to investigate for changes in the gene expression in the specific regions of the brain associated with addiction to METH in rats. We examined the various areas of the central nervous system (striatum, hippocampus, prefrontal cortex) for signs of impairment in postnatal day 80 in experimental rats, whose mothers had been administered METH (5 mg/kg/day) during the entire gestation period. Changes in the gene expression at the mRNA level were determined by two techniques, microarray and real-time PCR. Results of two microarray trials were evaluated by LIMMA analysis. The first microarray trial detected either up-regulated or down-regulated expression of 2189 genes in the striatum; the second microarray trial detected either up-regulated or down-regulated expression of 1344 genes in the hippocampus of prenatally METH-exposed rats. We examined the expression of 10 genes using the real-time PCR technique. Differences in the gene expression were counted by the Mann–Whitney *U*-test. Significant changes were observed in the cocaine- and amphetamine-regulated transcript prepropeptide, tachykinin receptor 3, dopamine receptor D3 gene expression in the striatum regions, in the glucocorticoid nuclear receptor Nr3c1 gene expression in the prefrontal cortex and in the carboxylesterase 2 gene expression in the hippocampus of prenatally METH-exposed rats. The microarray technique also detected up-regulated expression of trace amine-associated receptor 7 h gene in the hippocampus of prenatally METH-exposed rats. We have identified susceptible genes; candidates for the study of an impairment related to methamphetamine addiction in the specific regions of the brain.

## Highlights

- The microarray technique detected either up-regulated or down-regulated expression of 2189 genes in the striatum of prenatally METH-exposed rats.
- The microarray technique detected either up-regulated or down-regulated expression of 1344 genes in the hippocampus of prenatally METH-exposed rats.
- We examined the expression of 10 genes using the real-time PCR technique.
- Prenatally administered METH significantly alters the expression of the glucocorticoid nuclear receptor Nr3c1 mRNA within prefrontal cortex of rats.
- Prenatally administered METH significantly alters the expression of the cocaine- and amphetamine-regulated transcript prepropeptide, the tachykinin receptor 3 and dopamine receptor D3 mRNA within striatum of rats.
- Prenatally administered METH significantly alters the expression of the carboxylesterase 2 mRNA within the hippocampus of rats.
- Prenatally administered METH alters the expression of the trace amine-associated receptor mRNA within hippocampus of rats
- Both techniques, microarray and real-time PCR, showed altered expression of the tachykinin receptor 3 and dopamine receptor D3 mRNA within striatum of rats.

## Introduction

Methamphetamine (METH) is one of the most common “hard” drugs taken by pregnant women (Marwick, 2000). It is also one of the most frequently used illicit drugs in the Czechia, Middle and Eastern Europe (Vavřinková et al., 2001; Šlamberová, 2012). Statistics show that only 17% of female drug abusers in the United States were primary METH users, but 38% had used it during pregnancy, because some drug-abused women replace other drugs for METH during pregnancy for its anorectic effect (Marwick, 2000). METH is a powerfully addictive stimulant that metabolizes slowly resulting in the drug having a long-lasting high (8 to 24 h) (Marwick, 2000). This might be the reason that makes it so popular. The National Institute of Drug Abuse (NIDA; part of NIH) in the United States decided to increase funding for studies testing the effects of METH administration during pregnancy. Research concerning the long-term effects of prenatal METH exposure is still in its infancy. Our laboratory specializes in investigating the effects of drugs (especially METH) on rat mothers and their progeny.

METH is an indirect agonist of monoamines, which works by disrupting multiple presynaptic and synaptic processes. The resultant increases in dopamine, norepinephrine, and serotonin contribute to its properties as well as inducing other central and peripheral effects (Thompson et al., 2009; Lloyd et al., 2013). In low doses, METH can elevate mood and increase alertness, concentration and energy. At higher doses, it can induce psychosis, breakdown of skeletal muscle, seizures and bleeding in the brain. Chronic, high-dose use can precipitate unpredictable and rapid mood swings, prominent delusions and violent behavior. Chronic abuse is associated with serious health complications including deficits in attention, memory, and executive functions in humans (Cadet and Krasnova, 2009). METH is known to have a high addiction liability (i.e., compulsive METH use) and dependence liability (i.e., withdrawal symptoms occur when METH use ceases) (ChEBI, 2018; Wikipedia contributors, 2019b). METH travels through the bloodstream to the brain, where it readily crosses the blood-brain barrier (Dattel, 1990; Burchfield et al., 1991). The similarity of the chemical structure of METH to monoamine neurotransmitters (dopamine and noradrenaline) determines its mechanism of action (Sulzer et al., 2005; Heal et al., 2013, Rambousek et al., 2014). METH affects the function and trafficking of DAT. It leads to increased release of dopamine from presynaptic terminals, as well as synaptic vesicles (Sulzer et al., 1995). Methamphetamine interacts with Taar1 receptors and this interaction results in inhibition of dopamine uptake, induction of dopamine efflux, and promotion of dopamine transporter internalization. TAAR1 mediates methamphetamine-induced regulation of dopamine transporter function and trafficking in brain striatal synaptosomes (Xie and Miller, 2009; Rutigliano et al., 2018). In Taar1-knock out mice sensitivity to amphetamine and methamphetamine was exacerbated (Lindemann et al., 2008; Achat-Mendes et al., 2012; Rutigliano et al., 2018). The neuropsychiatric complications might, in part, be related to drug-induced neurotoxic effects, which include damage to dopaminergic and serotonergic terminals, neuronal apoptosis, as well as activated astroglial and microglial cells in the brain (Cadet and Krasnova, 2009).

METH present in a drug-abusing women’s bloodstream, can pass through the placenta to a fetus and can also be secreted into breast milk (Winslow et al., 2007). Amphetamines have vasoactive effects, which result in the restrictions of the supply of nutrients to a developing fetus, causing the anorectic symptoms often reported (Winslow et al., 2007). Infants born from METH-abusing mothers were found to have significant growth reductions (Dixon and Bejar, 1989; Plessinger, 1998; Cui et al., 2006), cleft palate, cardiac defects (Plessinger, 1998; Cui et al., 2006), aggressive behavior (Eriksson et al., 1989; Cui et al., 2006), peer-related problems (Eriksson and Zetterstrom, 1994; Cui et al., 2006) and learning problems (Cernerud et al., 1996; Cui et al., 2006). Matera and colleagues reported severe brain malformations in a human infant exposed to amphetamine *in utero*, such as exencephaly, anomalies of gyria, and hemorrhage (Matera et al., 1968; Cui et al., 2006). Human infants exposed to methamphetamine *in utero* have tended to show a significant decrease in head circumference (Little et al., 1988; Dixon and Bejar, 1989; Cui et al., 2006; Winslow et al., 2007). Non-invasive studies of the brain using Echo or MRI have revealed intracranial hemorrhage (Dixon and Bejar, 1989; Cui et al., 2006) and volume decrease of subcortical area (Chang et al., 2004; Cui et al., 2006). The effects of METH-administered prenatally in clinical studies are summarized in Table 1.

**TABLE 1:** The list of published results of clinical trials with METH-administered prenatally.

**Etiopatogenetic factor**	**Effect**	**Author**
Amphetamine administration during pregnancy	Brain malformations, such as exencephaly, anomalies of gyria, and hemorrhage	Matera et al., 1968; Cui et al., 2006
METH and amphetamine administration during pregnancy	At birth, 1, and 4 years: the mean weight, height, and head circumference below average Age 10: females, but not males, significantly shorter and lighter At 14–15 years: achievement in mathematics, Swedish language, and sports were statistically low	Eriksson et al., 1978; Cernerud et al., 1996; Wouldes et al., 2004
	An increased rate of premature delivery and placental abruption	Eriksson et al., 1978, 1981; Wouldes et al., 2004
METH, amphetamine, cocaine administration during pregnancy	Cleft lip and palate, heart defects, low birth weight, small head circumference, dead fetus, biliary atresia, undescended testicles, low body fat, premature birth	Eriksson et al., 1978; Oro and Dixon, 1987; Little et al., 1988; Dixon and Bejar, 1989; Cui et al., 2006
Perinatal administration of METH and cocaine	Tachycardia, bradycardia, abnormal sleep, tremors	Oro and Dixon, 1987
METH administration during pregnancy	Decreased fetal-growth, no congenital anomalies	Little et al., 1988; Cui et al., 2006
Drug administration during pregnancy	Delayed development	van Baar et al., 1989
Maternal methamphetamine abuse	Fetal and infant death	Stewart and Meeker, 1997
METH and amphetamine administration during pregnancy	At 8 years: significant correlation between amount and duration of exposure prenatally and aggressive behavior and social adjustment	Billing et al., 1994; Eriksson and Zetterstrom, 1994; Wouldes et al., 2004
METH and amphetamine administration during pregnancy	Cleft, cardiac anomalies and fetal growth retardation	Plessinger, 1998; Wouldes et al., 2004; Cui et al., 2006
METH administration during pregnancy	Increased muscle tone, tremors, irritability, irregular sleep, impaired adaptability to stress	Wouldes et al., 2004
METH administration during pregnancy	Smaller subcortical volumes and associated neurocognitive deficits, neurotoxicity to the developing brain	Chang et al., 2004; Cui et al., 2006
Pre-clinical trial: prenatal methamphetamine exposure	Reduced dopamine transporter density and reduced dopamine D2 receptors in the striatum, lower levels of serotonergic transporter density and vesicular monoamine transporter across striatal sub regions	Chang et al., 2007
METH administration during pregnancy	Anorectic symptoms	Winslow et al., 2007
METH administration during pregnancy	Subtle reductions in spatial performance in the Memory Island test	Piper et al., 2011
METH administration during pregnancy	Increased emotional reactivity and anxious/depressed problems at ages 3 and 5 years; Attention-deficit/ hyperactivity disorder problems by age 5 years; Attention problems and withdrawn behavior at ages 3 and 5 years	LaGasse et al., 2012
Prenatal METH and tobacco exposure	Significantly reduced caudate nucleus volumes and cortical thickness increases in perisylvian and orbital-frontal cortices; subtle attentional deficits	Derauf et al., 2012
Prenatal exposure to METH	Significantly higher cognitive problems subscale scores than comparisons and were 2.8 times more likely to have cognitive problems scores that were above average	Diaz et al., 2014

Experimental studies demonstrated that administration of METH to pregnant rats leads to increased likelihood of abortion and maternal death (Acuff-Smith et al., 1992; Matějovská et al., 2014). Pregnant rats who receive METH have a shorter gestation period and have fewer litters of young than control mothers (Martin, 1975; Martin et al., 1976). METH-administered prenatally increases infant mortality, reduces weight gain, delays development and slows down reflexes (Martin et al., 1976; Cho et al., 1991; Acuff-Smith et al., 1996; Matějovská et al., 2014). Studies from our laboratory and others demonstrated that prenatal or neonatal exposure to METH seriously impairs the behavior of adult rats and their sensitivity to drugs (Šlamberová, 2012). Also, our studies demonstrated that maternal addiction during gestation and/or lactation periods impairs maternal behavior, which in turn has adverse effects on pup development (Šlamberová et al., 2005a,b, 2006). Such impairment caused by prenatal METH exposure can be partially suppressed by cross-fostering with control rats, demonstrating the positive effect of good postnatal foster care (Hrubá et al., 2009, 2010; Pometlová et al., 2009). On the other hand, maternal separation induces social stress and maternal physical stress, aggravating cognitive impairment and responses to stressors in the exposed offspring (Holubová et al., 2016, 2018). Thus, it seems that social factors, such as bad or good maternal care or maternal stress, can have significant influence on the development of rat offspring exposed to METH during gestation. In addition, one of our studies found that drug exposure during gestation may even influence second generation pups (Šlamberová et al., 2005d, 2007).

There are several brain structures that are associated with drug addiction. The most important are mesolimbic, and mesocortical pathways (Robinson and Becker, 1986) which includes the striatum, hippocampus and prefrontal cortex. Striatum coordinates multiple aspects of cognition, including both motor and action planning, decision-making, motivation, reinforcement, and reward perception (Yager et al., 2015). The hippocampus plays important roles in the consolidation of information from short-term memory to long-term memory, and in spatial memory that enables navigation (Anderson et al., 2007). The prefrontal cortex has been implicated in planning complex cognitive behavior, personality expression, decision making, and moderating social behavior (Yang and Raine, 2009). Various studies have found that prenatal exposure to psychostimulants results in enhanced responsiveness of mesostriatal monoaminergic projections (Bubeníková-Valešová et al., 2009; Fujáková-Lipski et al., 2017). Given that our previous studies demonstrated changes in locomotor activity, cognition as well as social behavior (Fialová et al., 2015; Šlamberová et al., 2015), types of behavior that are associated with the above mentioned brain structures, the aim of the present study was to identify candidates for changes in the gene expression in the striatum, hippocampus and prefrontal cortex in adult rats in PD 80, whose mothers had been administered METH (5 mg/kg/day) during the entire gestation period.

## Materials and Methods

### Animals and Housing

The procedures for animal experimentation utilized in this study were reviewed and approved by the Committee for Protection of Experimental Animals of Third faculty of Medicine and by Departmental Committee of Ministry of Education, Youth, and Sports and are in agreement with the Czech Government Requirements under the Policy of Humans Care of Laboratory Animals (no. 246/1992) and also comply with the regulations of the Ministry of Agriculture of the Czech Republic (no. 311/1997). Adult male (300–400 g) and female (250–300 g) Wistar rats from Charles River Laboratories International, Inc., were delivered by VELAZ (Prague, Czechia) and housed 4–5 per cage in a temperature-controlled (22–24.8°C) colony room with a standard 12 h light/dark cycle, lights on at 06.00 h. Animals were left undisturbed for 1 week with food and water *ad libitum*. After the acclimation period, the females were weighed and smeared using vaginal lavage to determine the phase of the estrous cycle. Females at the onset of the estrous phase of the estrous cycle were housed with sexually mature males for overnight (1 pair per cage) (Šlamberová et al., 2005a,b,c,d;Schutová et al., 2012). On the following day, the females were smeared and returned to their previous home cages. The fertilization was assigned as Day 1 of gestation based on the presence of sperm in the vaginal smear. On Day 21 of gestation, the females were removed from the group cages and placed into maternity cages (1 female per cage). The day of the delivery was counted as PD 0. The number of pups in each litter was adjusted to 12. Whenever possible, the same numbers of male and female pups were kept in each litter. To avoid litter bias, pups were cross-fostered on PD 1 so that one mother usually raised three male and three female pups of her own and three male and three female pups from another mother (see below). On PD 21, animals were weaned and housed in groups separated by sex. The light/dark cycle of the animals was reversed with lights on at 18.00 h. Animals were left undisturbed until adulthood (Schutová et al., 2012).

### Drug Administration and Experimental Groups

For prenatal exposure, the pregnant dams were divided into two groups: the METH group and the SAL group. The females from the METH group were administered D-methamphetamine HCl (Sigma Aldrich, Czechia) at a dose of 5 mg/kg/day and volume of 1 ml/kg/day s.c. for the duration of the gestation period (i.e., from the first to the last day of gestation) (Šlamberová et al., 2005a,b,d,^2006^; Schutová et al., 2012). This METH dose was chosen because it results in similar fetal brain drug concentrations to those found in human infants of METH-abusing women (Acuff-Smith et al., 1996; Schutová et al., 2012). This dose is also the standard dose used in our experiments (Šlamberová et al., 2005a,b,d, 2006, 2007; Schutová et al., 2012). The females from the SAL group were administered 0.9% NaCl solution s.c. at the same time and in the same volume as METH. On PD 1, the offspring were marked according to the prenatal exposure using the intradermal application of black India ink; the METH group of prenatally affected offspring was marked in the left foot pad, and the SAL group of prenatally affected offspring was marked in the right foot pad.

### Study Subjects

Before we started the experiment in PD 80, the experimental animals (8 METH and 8 SAL rats) were weighed as were the parts of the brains from which the RNA was isolated. The isolation of total RNA (mixture of mRNA, tRNA, and rRNA) from the snap-frozen tissue was obtained in accordance with the published procedure by Zhu Shirley: Total RNA isolation and purification for microarray (Gilks et al., 2005). The cerebral parts of experimental rats, striatum, hippocampus and prefrontal cortex, were obtained and stored in TRI reagent (Sigma-Aldrich, United States). The tissue was homogenized, centrifuged, washed with chloroform, and precipitated in isopropanol. Total RNA from the striatum, hippocampus and prefrontal cortex was used in the microarray and real-time PCR techniques.

### Microarray Technique

The microarray technique was carried out using Genechip Rat Gene 2.0 ST Array (Affymetrix, United States) in the Genomics and Bioinformatics department, Institute of Molecular Genetics of the ASCR (Prague, Czechia). The procedure was carried out twice. Because of the limited resource only 6 properly isolated samples from striatum (from 3 METH and from 3 SAL rats) were examined to distinguish the mRNA expression level of 29489 genes by the microarray technique in the first trial. Also, because of the limited resource only 6 properly isolated samples from the hippocampus (from 3 METH and from 3 SAL rats) were examined to distinguish the mRNA expression level of 29489 genes by the microarray technique in the second trial. Results of both examinations were evaluated by LIMMA analysis in the Genomics and Bioinformatics department, Institute of Molecular Genetics of the ASCR (Prague, Czechia).

### Real-Time PCR Technique

Total RNA (70 ng) from 8 METH and 8 SAL rats was processed using TaqMan Gene Expression Assays (ThermoFisher Scientific, United States) and TaqMan Gene Expression Master Mix (ThermoFisher Scientific, United States) by real-time PCR in the 7500 Fast Real-Time PCR System (ThermoFisher Scientific, United States). Together 6 genes, Gapdh, Pgk1, 18sRNA, B2m, Rps6, PPIA (Bernaudin et al., 2002; Hori et al., 2005; Shilling et al., 2006; Romanowski et al., 2007; Langnaese et al., 2008; Gubern et al., 2009) were assessed for their potential as being suitable reference genes in all three types of tissue. Reference genes, Rps6 and B2m were chosen according to the best reproducible results observed for gene expression in all three of tissues (striatum, hippocampus, prefrontal cortex), and taking into account the published results of another scientific group (Shilling et al., 2006). A quantification of gene expression was calculated by the Pfaffl method (Pfaffl, 2001; Šmehilová, 2011); exponential amplifications factors of Assays were equal to 1 (ThermoFisher Scientific, United States). Differences were counted by the Mann–Whitney *U*-test.

### Gene Anthology

The first group of genes (Table 2), which was examined using real-time PCR, includes Bdnf, nuclear receptor subfamily 3 group C member 1 (Nr3c1), Esr1, insulin-like growth factor I receptor (Igfr1), and olfactory factor 522 (Olr522). The genes were chosen based on data published by various scientific groups (Middaugh, 1989; Cernerud et al., 1996; Wouldes et al., 2004; Šlamberová and Rokyta, 2005; Chang et al., 2007; Ago et al., 2009; Schutová et al., 2008, 2009, 2012; Lloyd et al., 2013; Macúchová et al., 2013, 2014; Leung et al., 2014; Šlamberová et al., 2014; Ren et al., 2016, 2017). The second group of genes (Table 3), which was examined using real-time PCR, includes Tacr3, dopamine receptor D3 (Drd3), Cartpt, Rps6ka a Foxp2 genes. The genes were chosen based on our results from the microarray technique and in accordance with data published by other scientific groups (Paris and Lorens, 1989; Gecz and Mulley, 2000; Couceyro et al., 2005; Le Foll et al., 2005; Krasnova et al., 2017). Together, the expression of 10 genes was examined using the real-time PCR technique.

**TABLE 2:** The first group of examined genes with the description of their function according to the NCBI gene database.

**Gene**	**Name**	**Function (NCBI gene database)**	**Published results of other scientific groups**
OLR522	Olfactory receptor 522	Interaction with odorant molecules in the nose; initiation of a neuronal response that triggers the perception of a smell	
Bdnf	Brain-derived neurotrophic factor	Plays a role in the development of hippocampal long term potentiation; involved in regulation of synaptic plasticity	Brain-derived neurotrophic factor is involved in the METH dependence (Ren et al., 2016, 2017).
Nr3c1	Nuclear receptor subfamily 3, group C, member 1	Glucocorticoid receptor that binds and activates hormone-dependent transcriptional enhancers	Glucocorticoid receptors are involved in METH-induced hyperactivity (Ago et al., 2009).
Esr1	Estrogen receptor 1	Acts as a transcriptional activator when bound to estrogen; may play a role in myocardial regulation	The gene ESR1 may play a role in the pathophysiology of methamphetamine induced psychosis patients (Kishi et al., 2009)
Igf1r	Insulin-like growth factor 1 receptor	Involved in induction of cell cycle progression and survival in many cell types	Insulin like growth factor binding protein 5 is involved in METH-induced apoptosis (Leung et al., 2014).

**TABLE 3:** The second group of examined genes with the description of their function according to the NCBI gene database.

**Gene**	**Name**	**Function (NCBI gene database)**	**Published results of other scientific groups**
Foxp2	Forkhead box P2	Proper development of speech and language regions of the brain during embryogenesis	The Forkhead box P2 gene is a candidate to study cognitive functions (Gecz and Mulley, 2000)
Rps6ka3	Ribosomal protein S6 kinase A3	Controlling cell growth and differentiation	
Cartpt	Cocaine- and Amphetamine-Regulated Transcript prepropeptide	Neuronal protein that may play a role in brain development and may be regulated by testosterone	An upregulation of Cartpt gene in the striatum of shock-resistant rats related to addiction to METH (Krasnova et al., 2017)
Tacr3	Tachykinin receptor 3	Receptor for tachykinin neuromedin K (neurokinin B); couples with G protein that activates the phosphatidylinositol-calcium second messenger system	The sequence variations in tachykinin receptor 3 are associated with alcohol and cocaine addiction (Foroud et al., 2008).
Drd3	Dopamine receptor D3	May be involved in facilitating the effects of antipsychotic drugs and drug treatments for Parkinson’s disease	A single cocaine exposure increases Bdnf and D3 receptor expression (Le Foll et al., 2005).

## Results

The weights of SAL animals ranged from 285 to 360 g with a median of 310,5 g. The weights of METH animals ranged from 298 to 350 g with the median of 330 g. The animals were divided into boxes of four (1–4, 5–8).

### Results of Microarray Trials

#### Results of the First Microarray Trial in the Striatum

The mRNA expression (cDNA) of 29489 genes was examined in 6 striatum samples: three samples from the prenatally METH-exposed rats and three samples from the prenatally SAL-exposed rats. The expression of approximately 2189 genes was either up-regulated or down-regulated (*p* < 0.05) in the striatum, however, the differences were very low. The borderline significant difference (*p* < 0.05 and logFC > 1) between METH and SAM samples was observed in the case of Drd3 and Tacr3 genes (Table 4) (Zoubková, 2019a). The significant difference (*p* < 0.05 and logFC > 1) between METH and SAM samples was observed in the case of Cartpt gene, olfactory receptor 522 and 1448 genes (Table 4) (Zoubková, 2019a). The non-significant difference between METH and SAM samples was observed in the case of Foxp2 and Rps6ka3 genes (Table 4) (Zoubková, 2019a).

**TABLE 4:** The most relevant results of the first microarray trial in the striatum.

**Gene**	**GENENAME**	**logFC**	***p-*value**
Olr522	Olfactory receptor 522	−1.45	4.9e-06
Foxp2	Forkhead box P2	0.36	0.011
Rps6ka3	Ribosomal protein S6 kinase polypeptide 3	0.29	0.017
Cartpt	Cocaine- and amphetamine-regulated transcript prepropeptide	1.09	0.0021
Tacr3	Tachykinin receptor 3	0.88	0.00021
Drd3	Dopamine receptor 3	0.94	0.00041
Olr1448	Olfactory receptor 1448	−1.06	3.6e-05

#### Results of the Second Microarray the Trial in the Hippocampus

The mRNA expression (cDNA) of 29489 genes was examined in 6 hippocampus samples: three samples from the prenatally METH-exposed rats and three samples from the prenatally SAL-exposed rats. The expression of approximately 1344 genes was either up-regulated or down-regulated (*p* < 0.05) in the hippocampus, however, the differences were very low. The borderline significant difference (*p* < 0.05 and logFC > 1) between METH and SAM samples was observed in the case of Taar7h gene. The significant difference *(p* < 0.05 and logFC > 1) between METH and SAM samples was observed in the case of carboxylesterase 2i (Ces2) and olfactory receptor 1726 genes (Table 5). The non-significant difference between METH and SAM samples was observed in the case of Tacr3 gene (Zoubková, 2019b).

**TABLE 5:** The most relevant results of the second microarray trial in the hippocampus.

**SYMBOL**	**GENENAME**	**logFC**	***p*-value**
Ces2i	Carboxylesterase 2	−1.04	0.006
Olr1726	Olfactory receptor 1726	−1.17	0.0044
Taar7h	Trace amine-associated receptor 7 h	0.82	0.00077
Tacr3	Tachykinin receptor 3	0.19	0.039

#### Results of Real-Time PCR

For each of the tissue types (striatum, hippocampus and prefrontal cortex) the expression of chosen genes was examined using real-time PCR with TaqMan^®^ Gene Expression Assays (ThermoFisher Scientific, United States). The measured values were normalized to the measured values of two reference genes, B2m and Rps6, and quantified by the Pfaffl method (calculation of ratio of target to reference gene). Significant differences between METH and SAM samples were evaluated by the Mann–Whitney *U*-test (Supplementary Table S1).

#### Results of Real-Time PCR in the Prefrontal Cortex

**FIGURE 1:**
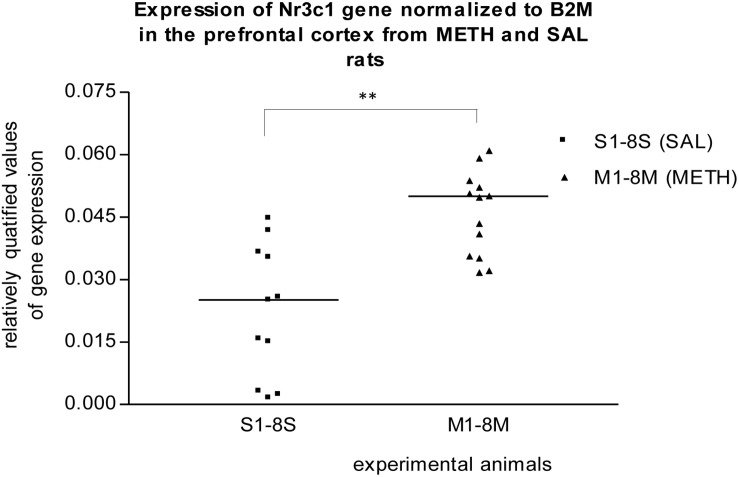
Significant difference (*p* ≤ 0.01) in the expression of Nr3c1 gene between METH and SAL samples in the prefrontal cortex. The results of the Nr3c1 gene expression were normalized to the results of the expression of the reference B2m gene. The values of METH rats are marked as M and the values of SAL rats are marked as S. The values are presented with a median. The number of values is higher than 8 M and 8 S, respectively 8 METH and 8 SAL, because we also used the remaining samples from the pilot study (S1–S3, M1–M5).

In the prefrontal cortex of prenatally METH-exposed rats, the mRNA expression of the Nr3c1 gene was significantly (*p* ≤ 0.01) increased compared to that in prenatally SAL-exposed rats (Figure 1). The measured values were normalized to the measured values of the reference B2m gene. However, the increase was not detected, when the measured values were normalized to the measured values of the reference Rps6 gene. The mRNA expression of the Foxp2 gene of prenatally METH-exposed rats was significantly (*p* ≤ 0.05) decreased compared to that in prenatally SAL-exposed rats in the prefrontal cortex, when the measured values were normalized to the measured values of the reference Rps6 gene. However, no difference was observed, when the measured values were normalized to the measured values of the reference B2m gene.

#### Results of Real-Time PCR in the Striatum

**FIGURE 2:**
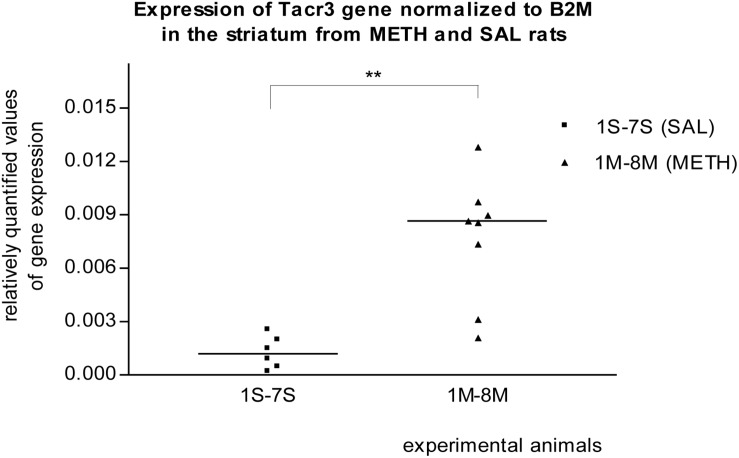
Significant difference (*p* ≤ 0.01) in the expression of the Tacr3 gene between METH and SAL samples in the striatum. The results of the Tacr3 gene expression were normalized to the results of the expression of the reference B2m gene. The values of METH rats are marked as M and the values of SAL rats are marked as S. The values are presented with a median.

**FIGURE 3:**
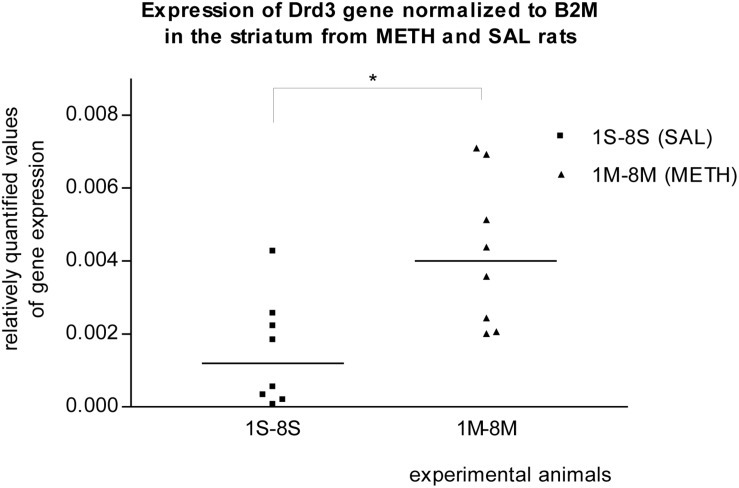
Significant difference (*p* ≤ 0.05) in the expression of the dopamine D3 receptor (Drd3) gene between METH and SAL samples in the striatum. The results of the Drd3 gene expression were normalized to the results of the expression of the reference B2m gene. The values of METH rats are marked as M and the values of SAL rats are marked as S. The values are presented with a median.

In the striatum of prenatally METH-exposed rats, the mRNA expression of Tacr3 gene (*p* < 0.01) and dopamine receptor D3 gene (*p* < 0.05) were significantly increased compared to that in prenatally SAL-exposed rats. The measured values were normalized to the measured values of the reference B2m gene (Figures 2, 3). No increase however was observed, when the measured values were normalized to the measured values of the reference Rps6 gene.

#### Results of Real-Time PCR in the Hippocampus

No differences in the mRNA expression were observed for any of the genes between prenatally METH-exposed rats and prenatally SAL-exposed rats in the hippocampus, even if the measured values were normalized to the measured values of either Rps6 or B2m reference genes.

## Discussion

Using a real-time PCR technique we examined changes in gene expression in the striatum, hippocampus, and prefrontal cortex. Using microarray we examined the changes in gene expression in the striatum and hippocampus of prenatally METH-exposed rats. Despite our expectations, we found only three genes in the striatum from approximately 2189 up-regulated or down-regulated genes, and found only two genes in the hippocampus from approximately 1344 up-regulated or down-regulated genes, whose mRNA expression were significantly altered (*p* < 0.05 and logFC > 1). In addition, according to our previous studies, particularly the striatum and hippocampus are structures that demonstrated the impairment associated with METH-induced behavioral changes (Bubeníková-Valešová et al., 2009; Fialová et al., 2015; Fujáková-Lipski et al., 2017). For that reason we did not perform the third microarray trial with the samples from the prefrontal cortex, where we did not expect many changes.

### Olfactory Receptors

The microarray technique demonstrated significant decrease in the mRNA expression of the genes encoding the olfactory receptors 522 and 1448 in the striatum of prenatally METH-exposed rats. In the hippocampus of prenatally METH-exposed rats the microarray technique demonstrated significant decrease in the mRNA expression of the olfactory receptor 1726. Given that the olfactory receptors are only present in the Rattus norvegicus species (NCIB database) we did not further investigate the decreased mRNA of olfactory gene expression in the striatum and hippocampus, as such studies in rats would not contribute to our understanding of addiction to METH in humans.

### Reference Genes

Using real-time PCR 6 genes, Gapdh, Pgk1, 18sRNA, B2m, Rps6, and PPIA (Bernaudin et al., 2002; Hori et al., 2005; Shilling et al., 2006; Romanowski et al., 2007; Langnaese et al., 2008; Gubern et al., 2009) were assessed for their potential as being suitable reference genes in all three types of tissue. Reference genes, Rps6 and B2m, were chosen according to the best reproducible results observed for gene expression in all three types of tissues (striatum, hippocampus, prefrontal cortex).

#### Ribosomal Protein S6

Ribosomal Protein S6 is a major substrate of protein kinases in eukaryotic ribosomes (NCBI gene database). Recent studies concluded that cellular Ser/Thr kinase mTOR1 activation and rpS6 phosphorylation interrelated (Volovelsky et al., 2016; Huang et al., 2018). Activated mTORC1 complex initiates phosphorylation of rpS6 protein (Volovelsky et al., 2016; Huang et al., 2018), and METH decreases phosphorylation of mTORC1 and its downstream kinases (Liu et al., 2019). mTOR activation in the complexes with proteins, subsequent activation/phosphorylation of down-stream substrates (RSP6 protein) and METH-induced suppression of mTOR1 phosphorylation was also demonstrated in the nucleus accumbens (Huang et al., 2018). In summary, mTORC1 regulates cell proliferation through Rps6 phosphorylation and METH decreases phosphorylation of mTORC1. Given that RPS6 is related to METH metabolism, the results of analyses, where RPS6 is used as a reference gene, are questionable. Of note, the gene encoding Rps6ka3, which is Rps6 kinase, polypeptide 3, was one of 29489 up-regulated genes in the striatum in the first microarray trial.

#### Beta-2-Microglobulin

Beta-2-Microglobulin is a serum protein found in association with the major histocompatibility complex class I heavy chain on the surface of nearly all nucleated cells (Gussow et al., 1987; NCBI gene database). No differences in the expression of the B2m gene were observed in the striatum or in the hippocampus during the microarray trials. The B2m gene was therefore considered to be a suitable reference gene for use in the real-time PCR studies, when examining all three types of tissues in the prenatally METH-exposed rats.

The previous studies demonstrated that prenatal METH exposure can impair the development of the neonatal central nervous system (Williams et al., 2003; Šlamberová et al., 2006; Smith et al., 2008; Rambousek et al., 2014) and the developing brain seems to be one of the primary targets of developmental toxicities of amphetamine and methamphetamine (Thompson et al., 2009; Lloyd et al., 2013). There was evidence of oxidative DNA damage in embryonic and fetal brains of mice caused by amphetamines (Jeng et al., 2005; Wong et al., 2008). METH can be activated to free radical intermediates that initiate ROS formation and cause oxidative DNA damage in the brain (Jeng et al., 2006; Wong et al., 2008; Jeng and Wells, 2010; Ramkissoon and Wells, 2011). The damage leads to long-term postnatal neurodevelopmental deficits via a mechanism different from that underlying the neurodegeneration observed in METH-exposed adults (Jeng et al., 2005).

### Altered Gene Expression in the Striatum: Cartpt, Drd3, Tacr3

Prenatal METH exposure induces abnormalities in the brain’s structure and chemistry, especially in the striatum (Chang et al., 2007; Derauf et al., 2012). Won et al. (2001) described regional dependent distribution of METH in pup brains on gestational day 14 with the highest concentration in the striatum (Won et al., 2001; Rambousek et al., 2014). **Cart peptide** was detected in the rat nucleus accumbens and due to its distribution in the brain and its modulation of dopamine systems, may be involved in aspects of reward and drug abuse (Hubert and Kuhar, 2005; Kuhar et al., 2005). In our project, the microarray technique demonstrated a significant increase in the mRNA expression level of the gene coding Cart prepropeptide in the striatum of prenatally METH-exposed rats. The differences in mRNA expression between prenatally METH-exposed and prenatally SAL-exposed rats using microarray were very low. It appears that the dose 5 mg/kg/day of METH administered to the mothers during the gestation period might be too low to cause strong significant impairment in the development of the striatum, hippocampus and prefrontal cortex of the offspring. This may be the reason why we did not detect significant differences in the Cartpt gene expression using real-time PCR technique. However, when comparing our results to those of older studies, it must be pointed out that also acute administration of methamphetamine increased Cartpt mRNA level in the nucleus accumbens (Ogden et al., 2004; Salinas et al., 2006; Jean et al., 2007; Rogge et al., 2008). Kuhar and colleagues demonstrated that Cart peptides have a role in drug abuse by virtue of the fact that they are modulators of mesolimbic function (Kuhar et al., 2005). We can speculate that the mechanism of METH regulation of addiction is via the CREB phosphorylation pathway. METH self-administration causes enrichment of phosphorylated CREB (pCREB) (Krasnova et al., 2013) and CREB may activate Cartpt expression (Rogge et al., 2008), and biologically active Cart peptide plays a role in reward and addiction (Entrez PubMed, 2019a; GeneCards, 2019a).

It was previously reported that METH self-administration was associated with transcriptional changes in genes that regulate transcription, synaptic transmission, and synaptic plasticity in the dorsal striatum (Krasnova et al., 2013). These molecular changes are associated with the transition from recreational drug use to addiction (Volkow et al., 2001; Krasnova et al., 2013). **Dopamine Receptor D3** has the highest binding affinity for endogenous dopamine of all known dopamine receptors (Sokoloff et al., 1990; O’Brien and Gardner, 2005) and shows preferential neuroanatomical localization in the limbic area (Bouthenet et al., 1991; Le’vesque et al., 1992; Levant, 1998; Diaz et al., 2000; O’Brien and Gardner, 2005). In our real-time PCR studies, the expression of the Drd3 gene in the striatum of METH exposed rats appeared to be significantly (*p* ≤ 0.05) increased compared to that in SAL exposed rats. Borderline significant increased mRNA expression of dopamine receptor D3 gene in the striatum was also observed in the first microarray study. Le Foll et al. (2005) reported that a single administration of METH induces a transient increase in BDNF expression in the prefrontal cortex, associated with a long-lasting elevation in dopamine receptor D3 binding and mRNA levels in the nucleus accumbens (Le Foll et al., 2005; Sokoloff et al., 2006). From cocaine use receptors D3 appeared to be up-regulated in the nucleus accumbens (Staley and Mash, 1996; Segal et al., 1997; Neisewander et al., 2004; O’Brien and Gardner, 2005). Given that the effects of METH are believed to result primarily from the increased release of synthesized dopamine and inhibition of dopamine uptake (O’Brien and Gardner, 2005; Chen et al., 2014) and that elevated extracellular concentrations of dopamine lead to increased stimulation of dopaminergic receptors (Robinson and Becker, 1986; Pierce and Kumaresan, 2006; Rambousek et al., 2014) we could speculate that the basis of Drd3 contribution to the addiction to METH is in its sensitivity. A normalization of dopamine receptor D3 function may reduce vulnerability to relapse in psychostimulant abuse (Le Foll et al., 2005; Chen et al., 2014), and a blockade of the dopamine receptor D3 by an antagonist attenuated the incentive motivational effects of METH in rats and may have pharmacotherapeutic potential in the treatment of methamphetamine addiction (Heidbreder et al., 2005; Chen et al., 2014). **Tachykinin Receptor 3 gene** (NK-3 receptor) expression was significantly (*p* ≤ 0.01) increased in the striatum of METH exposed rats compared to that in SAL exposed rats in our real-time PCR studies. Borderline significant increased mRNA expression of the Tacr3 gene in striatum was also detected in the microarrays trial. Of further note, we observed that the expression of the Tacr3 gene was also non-significantly up-regulated in METH hippocampus samples in our second microarray study. This result provides new evidence of METH and Tacr3 relation or interaction. The previous findings described the relation between METH neurotoxicity and neurokinin-1 (NK-1) receptor. The striatal neurokinin-1 receptors modulated METH-induced production of nitric oxide (Wang and Angulo, 2011). METH induces pre- and post-synaptic damage in the striatum and can be prevented with pharmacological blockade of the NK-1 receptor (Yu et al., 2004; Tulloch et al., 2011). It was also previously reported that the NK-3 receptor (Tacr3) controls nucleus accumbens dopamine responses to cocaine in rats (Jocham et al., 2007; Barros et al., 2013) and to alcohol in humans (Foroud et al., 2008). In monkeys, repeated administration of cocaine induces a decrease in methylation of NK3 receptor gene (De Souza Silva et al., 2006; Silva et al., 2008) and the NK3 (Tacr3) antagonist blocks the behavioral effects of cocaine (Barros et al., 2013; Melamed et al., 2013). We can speculate that prenatal METH exposure, like cocaine, decreases the methylation status of the Tacr3 gene and increases the expression of the Tacr3 gene within striatum of rats. If the pharmacological blockage of tachykinin receptors 1 and 3 prevents the METH effects in the striatum, the Tacr3 and its gene is a suitable target for studying the addiction to METH.

### Altered Gene Expression in the Hippocampus: Ces2, Taar7h

Previous studies demonstrated that prenatal METH exposure may be neurotoxic to the developing brain; and prenatally METH-exposed children exhibited smaller hippocampus volumes compared with the control group correlated with poorer performance on sustained attention and delayed verbal memory (Chang et al., 2004). METH exposure early in life of mice caused sex- dependent impairments in object recognition, spatial learning, and memory in adulthood (Acevedo et al., 2007). In experimental rats, prenatal MA exposure caused an impairment of non-spatial memory (Šlamberová et al., 2014). Our microarray technique detected significant differences in the expression of the Ces2 **gene** in the hippocampus of prenatally METH- and SAL-exposed rats. Ces2 is a member of a large multigene family. The enzymes encoded by these genes are responsible for the hydrolysis of ester- and amide-bond-containing drugs such as cocaine and heroin. They also hydrolyze long-chain fatty acid esters and thioesters (Lian et al., 2018; Entrez PubMed, 2019b; GeneCards, 2019b; Wikipedia contributors, 2019a). The products of hydrolysis are generally more polar than the original ester resulting in an increase in water solubility, promoting renal elimination (Laizure et al., 2013). Mice and rats have plasma carboxylesterase activity, which scavenge many organophosphorus compounds, rendering animals more resistant to organophosphates (Duysen et al., 2011; Marrero-Rosado et al., 2018). The human carboxylesterases are located in the cytoplasm and endoplasmic reticulum of numerous tissues, but the greatest quantities are found in the liver and small intestine where they contribute significantly to the first-pass metabolic hydrolysis of substrate drugs (Imai et al., 2003, 2006; Ross and Crow, 2007; Taketani et al., 2007; Hatfield et al., 2011; Laizure et al., 2013). Our result provides evidence about prenatal METH-induced decrease in the expression of the Ces2 mRNA within the hippocampus of experimental rats. We suspect that the changes in the gene expression level in the hippocampus associated with addiction to METH would be more significant, if the prenatally METH-exposed experimental rats were subjected to stress-inducing conditions, such as social behavior, learning and memory tests. However, we can speculate that decreased expression of the Ces2 enzyme could contribute to such METH-induced impairment of hippocampus in rats. It is interesting to note that there is evidence of a clinically significant drug interaction of a carboxylesterase substrate and ethanol in humans. Ethanol-mediated inhibition of human Ces2 hydrolysis of cocaine was demonstrated in hepatic microsomes (Roberts et al., 1993; Laizure et al., 2013). The finding is an *in vitro* proof of an existence of metabolic process or signaling pathway, which could inhibit the function of Ces2 in mouse and human cells.

Our microarray technique detected up-regulated expression of Taar 7h gene in the hippocampus of prenatally METH-exposed rats. The specific function of Taar7h and other members of a family of Taars has not been determined yet (Rutigliano et al., 2018). Taars are widely distributed throughout peripheral and brain tissues. Some Taars appear to be olfactory receptors, at least in rodents (Liberles and Buck, 2006; Rutigliano et al., 2018). The most effects have been reported on the Taar1 receptor. The previous experiments revealed Taar1 expression in many distinct rodent CNS regions, namely, nucleus accumbens/olfactory tubercle, as well as limbic and monoaminergic areas, such as hippocampus (Borowsky et al., 2001; Bunzow et al., 2001; Rutigliano et al., 2018). Taar1 is a novel G protein-coupled receptor, which responds to a number of endogenous amines, as well as to number psychoactive drugs, i.e., amphetamines. Such sensitization effects of Taar1 to dopamine, serotonin and norepinephrine could account for its involvement in the rewarding effects of drugs of abuse. Accordingly, Taar1-knock out mice developed an earlier and longer-lasting methamphetamine induced conditioned place preference as compared to wild-type littermates (Achat-Mendes et al., 2012; Rutigliano et al., 2018). In addition, Taar1-knock out mice show increased locomotor response to amphetamine and, in the opposite case, the mice overexpressing Taar1 exhibited a lower response to the stimulating effects of amphetamine in terms of locomotor activity and monoamine release (Rutigliano et al., 2018). These findings are important in the understanding of our result of the increased expression of Taar7 gene and of the previous finding that adult female rats in diestrus and adult males postnatally exposed to METH via breast milk had decreased locomotion and exploratory behavior (Hrubá et al., 2012).

### Altered Expression in the Prefrontal Cortex: Foxp2, Nr3c1

Previous studies reported the prenatal METH-induced impairment of prefrontal cortex in experimental rats (Fujáková-Lipski et al., 2017; Hrebíčková et al., 2017) and in humans (Thompson et al., 2009). Mizoguchi and Yamada (2019) reported that patients addicted to methamphetamine exhibit impaired cognitive functions such as executive function, attention, social cognition, flexibility, and working memory (Mizoguchi and Yamada, 2019). From our studies we found that the expression of the **Foxp2 gene** was significantly (*p* ≤ 0.05) changed using real-time PCR and non-significantly changed using microarray technique in the prefrontal cortex of prenatally METH-exposed rats compared to the gene expression in prefrontal cortex of prenatally SAL-exposed rats. The data from real-time technique were normalized to the expression of the reference Rps6 gene. Given the fact that RPS6 phosphorylation is related to the metabolism of methamphetamine, the result from real-time PCR is questionable. However, the function of the neural learning system is modulated in the case of Foxp2 mutation (Chandrasekaran et al., 2015) and some cases of speech impairment were observed in prenatally METH-exposed children in some clinical trials (Cernerud et al., 1996; Wouldes et al., 2004).

In response to various stimuli, including stress, glucocorticoids coordinate metabolic, endocrine, immune, and nervous system responses (Liu et al., 1997; Tronche et al., 1999). In the brain, the glucocorticoid receptor has been thought to modulate emotional behavior, cognitive functions, and addictive states (Tronche et al., 1999). Liu et al. (1997) studied neuroendocrine responses to stress in rats and observed that adult rats, the offspring of mothers that exhibited more licking and grooming of pups during the first 10 days of life, exhibited increased glucocorticoid receptor mRNA expression in the hippocampus (Liu et al., 1997). From our studies, no apparent significant increases in gene expression were observed in the hippocampus of prenatally METH-exposed rats. Our results from the real-time PCR trials demonstrated that the expression of the **Nr3c1 gene** was significantly (*p* ≤ 0.01) increased in the prefrontal cortex of prenatally METH-exposed rats compared to that in prenatally SAL-exposed rats. Champagne et al. (2003) and Weaver et al. (2004) studied neuroendocrine responses to stress and Kosten et al. (2014) studied neuroendocrine responses to childhood abuse and both confirmed that variations in the maternal behavior of pup-licking are associated with the methylation status of the Nr3c1 gene (Champagne et al., 2003; Weaver et al., 2004; Kosten and Nielsen, 2014; Kosten et al., 2014). Future research should examine if prenatal METH exposure is able to alter the methylation status of the Nr3c1 gene. However, our finding is in accordance with findings reported by other scientific groups, that an increased motor activity and declined adaptability (Middaugh, 1989), change of behavior in an unknown environment, and an improvement of cognitive functions and anxiety (Schutová et al., 2008, 2009, 2012; Šlamberová et al., 2015) are associated to METH in gestation. The glucocorticoid nuclear receptor Nr3c1 and its gene are suitable targets for studying the changes associated with addiction to METH.

The differences in the mRNA expression between prenatally METH-exposed and prenatally SAL-exposed rats using both techniques were very low. It appears that the dose 5 mg/kg/day of METH administered to the mothers during the gestation period might be too low to cause strong significant impairment in the development of the striatum, hippocampus and prefrontal cortex of the offspring. We have not demonstrated robust changes in the gene expression levels of prenatally METH-exposed rats. We isolated total RNA from adult rats on PD 80. It is possible that the changes in the gene expression just after birth could have been more robust since postnatally induced permanent behavioral changes have an impact on gene expression in the brain and can dramatically change prenatally adjusted a gene expression scenario that can disappear in adulthood.

## Conclusion

The aims of our project were fulfilled. We have identified susceptible genes, candidates to the study of an impairment related to addiction to METH. Cartpt, Drd3, Tacr3 in the striatum and the Ces2 in the hippocampus have been found to be associated with addiction to METH. Future research should continue to confirm or refute our finding about an association between prenatal exposure to METH and the changes in the expression of Nr3c1 and Foxp2 genes in the prefrontal cortex. Further research is needed to confirm and investigate the novel finding of prenatally METH-induced decrease of Ces2 mRNA and prenatally METH-induced increase of Taar7h mRNA in the hippocampus. Our plan is to study the changes in the gene expression level in the striatum, hippocampus and prefrontal cortex of prenatally METH-exposed rats during the different stages of gestation. And in addition, we intend to study what genetic and epigenetic changes occur, when prenatally METH-exposed rats are subjected to varying stressful conditions.

## Data Availability

The datasets generated for this study can be found in Figshare, https://figshare.com/articles/Results_of_second_microarray_trial/8081120; https://figshare.com/articles/Results_of_first_microarray_trial/8081072.

## Ethics Statement

The study, the protocols and procedures with experimental animals were reviewed and approved by the Committee for Protection of Experimental Animals of Third faculty of Medicine and by Departmental Committee of Ministry of Education, Youth, and Sports. They are also in agreement with the Czech Government Requirements under the Policy of Humans Care of Laboratory Animals (no. 246/1992) and also comply with the regulations of the Ministry of Agriculture of the Czech Republic (no. 311/1997).

## Author Contributions

HZ isolated the RNA, chose the second group of the genes, carried out the real-time PCR experiments, analyzed data, and wrote the manuscript. AT participated in the real-time PCR experiments and in the data analysis. KN carried out the experiments with animals. MÈ participated in study design and coordination, chose the first group of the genes, and revised the manuscript. RŠ conceived the study, carried out the experiments with animals, and revised the manuscript. All authors approved the final version of the manuscript for submission.

## Conflict of Interest Statement

The authors declare that the research was conducted in the absence of any commercial or financial relationships that could be construed as a potential conflict of interest.

## Abbreviations

ASCR: Academy of Sciences of the Czech Republic
B2m: Beta-2-microglobulin
Bdnf: Brain-derived neurotrophic factor
Cartpt: Cocaine- And Amphetamine-Regulated Transcript prepropeptide
Ces2: Carboxylesterase 2
DAT: dopamine transporters
Drd3: Dopamine receptor D3
Esr1: Estrogen receptor 1
Foxp2: Forkhead box P2
Gapdh: Glyceraldehyde-3-phosphate dehydrogenase
Igfr1: Insulin-like growth factor 1
METH: Methamphetamine
mTORC1: mammalian target of rapamycin complex 1
NK-1: neurokinin 1
NK-3: neurokinin 3
Nr3c1: Nuclear receptor subfamily 3group c member 1
Olr1448: Olfactory receptor 1448
Olr1726: Olfactory receptor 1726
Olr522: Olfactory receptor 522
PD: Postnatal day
Pgk1: Phosphoglycerate kinase 1
PPIA: Peptidylprolyl isomerase A
Rps6: Ribosomal protein S6
Rps6ka3: Ribosomal protein S6 kinase A3
s.c.: Subcutaneously
SAL: Saline
Ser/Thr: Serine/threonine
Taar: trace amine-associated receptor
Taar7h: trace amine-associated receptor 7h
Tacr3: Tachykinin receptor 3.
